# Lost in transition 2.0: a long days’ journey towards continuity of care

**DOI:** 10.5334/ijic.860

**Published:** 2012-04-13

**Authors:** K. Viktoria Stein

**Affiliations:** Institute of Social Medicine, Centre for Public Health, Medical University of Vienna, Austria E-mail: katharina.v.stein@meduniwien.ac.at

It is said to be one of the key factors for the development of human societies and languages that people started to cooperate and coordinate their actions. The necessity to work together to reach a common goal, whether it be the slaying of a mammoth or the building of a spaceship, lead to the creation of communication, the invention of tools and the partition of tasks. Over the millennia, these vehicles have become ever more refined, detailed and specified which in turn triggered the demand for coordination, planning and attuned timing. And then there came health care.

I have often asked myself, why health care professionals and decision-makers alike believe that health care is so uniquely different from any other human endeavor that one just can’t generalise, standardise or organise it according to proven concepts of management. In any other sector it goes without saying that processes are defined and designed to lead the way through the host of departments, providers and professionals involved in creating a product or service to the satisfaction of the client. In health care, the establishment of patient pathways, medical guidelines and care networks adopt this role in order to create continuity in care. Essentially, this means defining the interfaces and their management: who does what, when and where. The idea is far from being revolutionary and successful enterprises have adhered to these principles since the erection of the pyramids. Still, when one speaks of transition management in health care it seems to many to be a novel concept.

One of the reasons may be that we are still not sure what continuity of care actually means and, more to the point, how we can reach it, as is described by Heaton et al. (http://www.ijic.org/index.php/ijic/article/view/794). Similar to ‘integrated care’ a myriad of concepts exist and discussions are still underway how transferable these are from one setting to another. As is often the case, when definitions are fuzzy and boundaries not well defined, one focuses on technicalities, losing one of the key ingredients to good quality service delivery out of sight: the client’s perspective. While patient participation is at the core of integrated care, examples are still rare of actual consideration of the patient’s perspective in the design and management of health care. It may not be necessary or even expedient to involve patients every step of the way, but it is certainly worthwhile to include their points of view in the continuum of care, as Breton et al. conclude (http://www.ijic.org/index.php/ijic/article/view/682).

The obstacles to continuity of care are usually found in weak or inexistent transitional management. There is little gained in high quality hospital care if the patient in need of assistance finds herself out on the street alone after discharge. The infamous ‘revolving door effect’, where patients are in and out of hospital on a regular basis, is the consequence of failed coordination between health and social care, primary and secondary care, doctors, nurses, formal and informal caregivers. The results are devastating for the patients’ quality of life, the professionals’ morale and the care systems’ resources. The loss of disability- or quality-adjusted life years due to discontinuity of care is considerable. The factors identified for poor transitional care for hip fracture patients by Toscan et al. (http://www.ijic.org/index.php/ijic/article/view/797) may be generalised to reasons for failed continuity of care: confusion with communication, unclear roles and responsibilities, competing ownership over service delivery and system constraints.

In today’s health and social systems, continuity of care must not be considered a decorative exercise, but an obligation towards the patients. This necessitates a more coordinated and cooperative mindset among the professionals involved and systems, which facilitate communication and foster change: ingredients, which are also essential for successful integrated care. Far from being a syllogism, continuity of care is hence a basic ingredient for integrated care and essential in the management of chronic diseases along with the patients who have them and the people involved in their care.

